# Supplementary material for: MetaQTL: a package of new computational methods for the meta-analysis of QTL mapping experiments
Source: BMC Bioinformatics. 2007 Feb 8;8:49. doi: 10.1186/1471-2105-8-49 (PMC1808479; doi:10.1186/1471-2105-8-49)
Supplement: Additional File 7 — MetaQTL Package : jar file and tutorial. This Zip archive contains both the MetaQTL JAR file and the files of the tutorial. [file 1471-2105-8-49-S7.zip › org.inra.metaqtl/doc/org/thalia/bio/util/ILGroupPosition.html]

ILGroupPosition


|  |  |  |
| --- | --- | --- |
| **Overview** \| **Package** \| **Class** \| **Use** \| **Tree** \| **Deprecated** \| **Index** \| **Help** | |  |
| **PREV CLASS**   **NEXT CLASS** | **FRAMES**    **NO FRAMES**     **All Classes** |  |
| SUMMARY: NESTED \| FIELD \| CONSTR \| METHOD | DETAIL: FIELD \| CONSTR \| METHOD |  |


---


## org.thalia.bio.util Interface ILGroupPosition

**All Known Implementing Classes:**: EstimatedLocusPosition, LGroupPosition, StaticLocusPosition

---

``` public interface ILGroupPosition ```

Class Description Here

**Author:**
:   Jean-Baptiste Veyrieras

---

| **Field Summary** | |
| --- | --- |
| `static int` | `ESTIMATED` |
| `static int` | `STATIC` |


| **Method Summary** | |
| --- | --- |
| `double` | `absolute()` |
| `int` | `getType()` |
| `double` | `standardError()` |

**Field Detail**

### STATIC

```
static final int STATIC
```

**See Also:**: Constant Field Values

---


### ESTIMATED

```
static final int ESTIMATED
```

**See Also:**: Constant Field Values


**Method Detail**

### absolute

```
double absolute()
```

---


### standardError

```
double standardError()
```

---


### getType

```
int getType()
```


---


|  |  |  |
| --- | --- | --- |
| **Overview** \| **Package** \| **Class** \| **Use** \| **Tree** \| **Deprecated** \| **Index** \| **Help** | |  |
| **PREV CLASS**   **NEXT CLASS** | **FRAMES**    **NO FRAMES**     **All Classes** |  |
| SUMMARY: NESTED \| FIELD \| CONSTR \| METHOD | DETAIL: FIELD \| CONSTR \| METHOD |  |


---
